# Supplementary material for: AlphaFold-Multimer predicts cross-kingdom interactions at the plant-pathogen interface
Source: Nat Commun. 2023 Sep 27;14:6040. doi: 10.1038/s41467-023-41721-9 (PMC10533508; doi:10.1038/s41467-023-41721-9)
Supplement: Supplementary file 1 — Supplementary Information [file 41467_2023_41721_MOESM1_ESM.pdf]

Supplementary Figures and Tables

Alphafold-multimer predicts cross-kingdom interactions at the plant-pathogen interface

Felix Homma, Jie Huang, Renier A. L. van der Hoorn

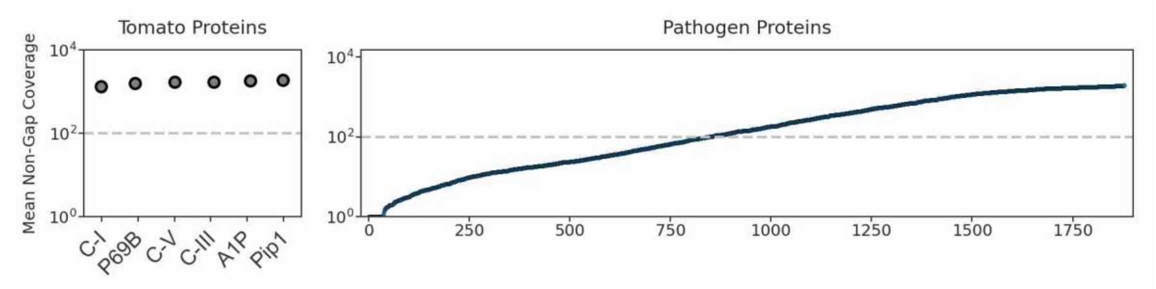

Supplementary Figure 1 MSA depth of SSPs and hydrolases used in Fig. 2.

The mean non-gap multiple sequence alignment (MSA) depth is shown for the tomato hydrolases (left) and the 1,879 SSPs (right), ranked by MSA depth. The dash line indicates the desired minimum of 100 MSA depth.

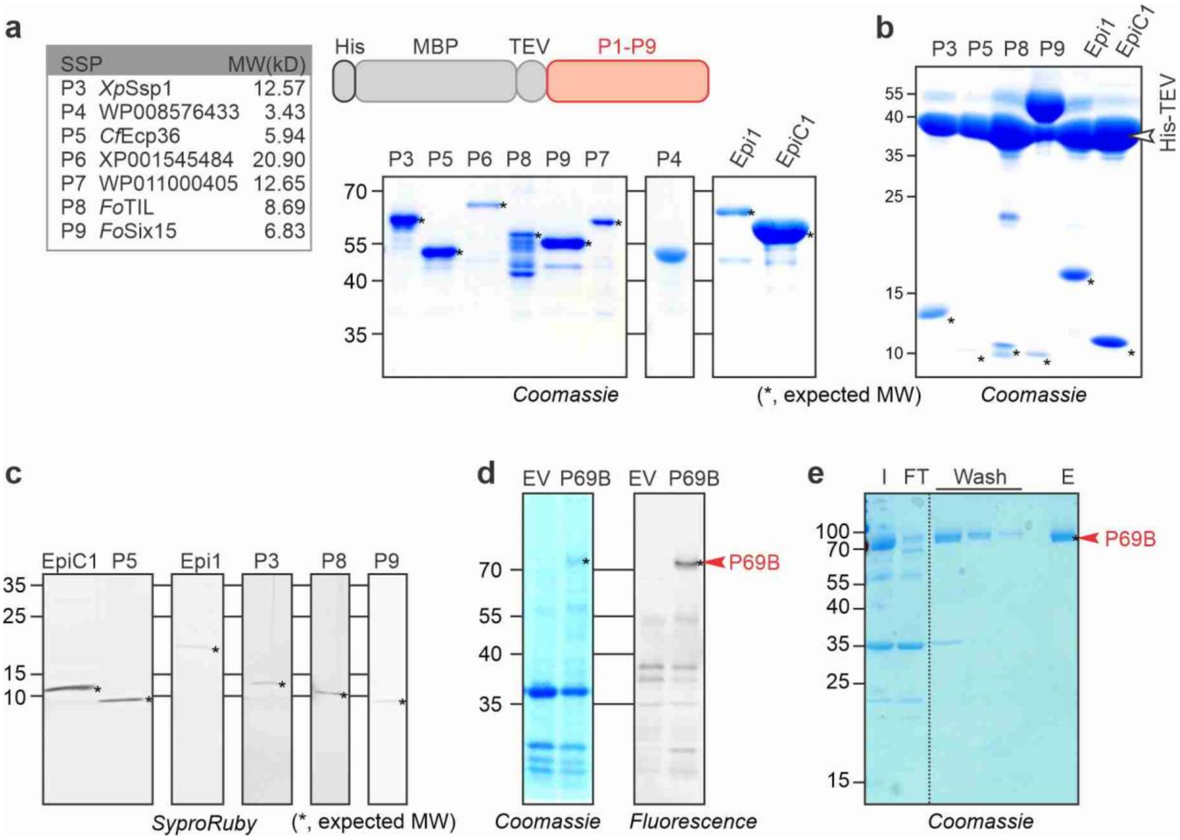

Supplementary Figure 2 Production of candidate inhibitors and P69B for inhibition assays. **a** Candidate inhibitors lacking their endogenous signal peptides were codon-optimized for expression in *E. coli* and cloned in-frame with a 6-His purification tag; the maltose binding protein (MBP) and the cleavage site of tobacco etch virus (TEV) protease. Fusion proteins were expressed in *E. coli* and purified on HisPur™ Ni-NTA resin and amylose resin, subsequently. Fusion proteins were detected at their predicted molecular weight (MW, *) by SDS-PAGE and Coomassie staining. Purification of candidate inhibitors was repeated at least once for each candidate independently. **b** Putative inhibitors were released from their purification tags upon incubation with His-TEV protease. This experiment was repeated at least once for each inhibitor independently. **c** Purified inhibitor candidates after removal of their purification tags and TEV proteins, stained with Sypro Ruby. This experiment was repeated at least once for each inhibitor independently. **d** P69B-His is efficiently expressed by agroinfiltration. P69B with a C-terminal His tag and an empty vector (EV) control were transiently expressed by agroinfiltration. Apoplastic fluids were isolated, labeled with FP-TAMRA, separated on SDS-PAGE and scanned for fluorescence (right) and stained with Coomassie (left). Expression of P69B was repeated at least twice to similar effect. **e** Purification of P69B-His on Ni-NTA. I, input sample; FT, flow through; E, eluate. The MW marker is presented in kDa on the left of the gels. Purification of P69B was repeated twice with similar results.

```
      1                                                                                                        100
lycop MGLLKILLVF IFCSFQWPTI QSNLETYIVH VESPESLVTT QSLLTDLGSY YLSFLPKTAT TISSSGNEEA ATMIYSYHNV MTGFAARLTA EQVKEMEKKH
pimpi MGLLKILLVF IFCSFQWPTI QSNLETYIVH VESPESLVTT QSSLTDLGSY YLSFLPKTAT TISSSGNEEA ATMIYSYHNV MTGFAARLTA EQVKEMEKKH
chees MGLLKILLVF IFCSFQWPTI QSNLETYIVH VESPESLVTT QSSLTDLGSY YLSFLPKTAT TISSSGNEEA ATMIYSYHNV MTGFAARLTA EQVKEMEKKH
habro MGLLKILLVF IFCSFPCPTI QSNFETYIVH VESPESLVTT LSSLTDLDSY YLSFLPKTAT TISSSGNEEA ATMIYSYHNV MTGFAARLTA EQVKEMEKKH
corne MGLLKILLVF IFCSFPWSTI QSNLETYIVH VESPESLVTT QSSLTDLDSY YLSFLPKTAT IISSSGNEEA ATMIYSYHNV MTGFAARLTA EQVKEMEKKH
neori MGLLKILLVF IFCSFPWPTI QSNLETYIVH VESPESLVTT QSSLTDLDSY YLSFLPKTAT TISSSGNEEA ATMIYSYHNV MTGFAARLTA EQVKEMEKKH
arcan MGLLKILLVF IFCSFQWPTI QSNLETYIVH VESPESLVTT QSSLTDLDSY YLSFLPKTAT TISSSGNEEA ATMIYSYHNV MTGFAARLTA EQVKEMEKKH
huayl MGLLKILLVF IFCSFQWPSI QSNLETYIVH VESPESLVTT QSSLTDLDSY YLSFLPKTAT TISSSGNEEA ATMIYSYHNV MTGFAARLTA EQVKEMEKKH
chile MGLLKILLVF IFCSFPWTTI QSNLETYIIH VESPESLVTT QSSLTDLDSY YLSFLPKTTT TISSSGNEEA ATMIYSYHNV MTGFAARLTA EQVKEMEKKH
penne MGLLKILLVF IFCSFPWPTI QSNLETYIVH VESPESLVTT QSSLTDLDSY YLSFLPKTTT TISSSGNEEA ATMIYSYHNV MTGFAARLTA EQVKEMEKKH
peruv MGLLKILLVF IFCSFPWPTI QSNLETYIVH VESPESLVTI QSSLTDLDSY YLSFLPKTTT TISSRGNEEA ATMIYSYHNV MTGFAARLTA EQVKEMEKKH

      101                                                                                                      200
lycop GFVSAQKQRI LSLHTTHTPS FLGLQQNMGV WKDSNYGKGV IIGVIDTGII PDHPSFSDVG MPPPPAKWKG VCESNFTNKC NNKLIGARSY QLGNGSPIDS
pimpi GFVSAQKQRI LSLHTTHTPS FLGLQQNMGV WKDSNYGKGV IIGVIDTGII PDHPSFSDVG MPPPPAKWKG VCESNFTNKC NNKLIGARSY QLGNGSPIDS
chees GFVSAQKQRI LSLHTTHTPS FLGLQQNMGI WKDSNYGKGV IIGVIDTGII PDHPSFSDVG MPPPPAKWKG VCESNFTNKC NNKLIGARSY QLGNGSPIDS
habro GFVSAQKQRM LSLHTTHTPS FLGLQQNMGV WKDSNYGKGV IIGVLDTGIL PDHPSFSDVG MPSPPAKWKG VCESNFTNKC NNKLIGARSY QLGNGSPIDS
corne GFVSAQKQRI LSLHTTHTPS FLGLQQNMGV WKDSNYGKGV IIGVIDTGII PDHPSFSDVG MPPPPAKWKG VCESNFTNNC NNKLIGARSY QLGNGSPIDS
neori GFVSAQKQRI LSLDTTHTPS FLGLQQNMGV WKDSNYGKGV IIGVIDTGII PDHPSFSDVG MPPPPAKWKG VCESNFTNKC NNKLIGARSY QLGNGSPIDS
arcan GFVSAQKQRI LSLDTTHTPS FLGLQQNMGV WKDSNYGKGV IIGVIDTGII PDHPSFSDVG MPPPPAKWKG VCESNFTNKC NNKLIGARSY QLGNGSPIDS
huayl GFVSAQKQRI LSLHTTHTPS FLGLQQNMGV WKDSNYGKGV IIGVIDTGII PDHPSFSDVG MPPPPAKWKG VCESNFTNKC NNKLIGARSY QLGNGSPIDS
chile GFVSAQKQRI LSLHTTHTPS FLGLQQNMGV WKDSNYGKGV IIGVLDTGII PDHPSFSDVG MPPPPAKWKG VCESNFTNKC NNKLIGARSY QLGNGSPIDS
penne GFVSAQKQRI LSLHTTHTPS FLGLQQNMGV WKDSNYGKGV IIGVIDTGII PDHPSFSDVG MPPPPAKWKG VCESNFTNNC NNKLIGARSY QLGNGSPIDS
peruv GFVSAQKQRI LSLHTTHTPS FLGLQQNMGV WKDSNYGKGV IIGVLDTGIL PDHPSFSDVG MPSPPAKWKG VCESNFTNKC NNKLIGARSY QLGNGSPIDS

      201                                                                                                      300
lycop IGHGTHTAST AAGAFVKGAN VYGNADGTAV GVAPLAHIAI YKVCNSVGCS ESDVLAAMDS AIDDGVDILS MSLSGGPIPF HRDNIAIGAY SATERGILVS
pimpi IGHGTHTAST AAGAFVKGAN VYGNADGTAV GVAPLAHIAI YKVCNSVGCS ESDVLAAMDS AIDDGVDILS MSLSGGPIPF HRDNIAIGAY SATERGILVS
chees IGHGTHTAST AAGAFVKGAN VYGNADGTAV GVAPLAHIAI YKVCNSVGCS ESDVLAAMDS AIDDGVDILS MSLSGGPIPF HRDNIAIGAY SATERGILVS
habro NGHGTHTAST AAGAFVKGAN VYGNADGTAV GVAPLAHIAI YKVCNSVGCS ESDILAAMDS AIDDGVDILS MSLGGGPIPF HKDNIAIGAY SATERGILVS
corne NGHGTHTAST AAGAFVKGAN VYGNANGTAV GVAPLAHIAI YKVCNSVGCS ESDVLAAMDS AIDDGVDILS MSLGGGPIPF HRDNIAIGAY SATERGILVS
neori NGHGTHTAST AAGAFVKGAN VYGNADGTAV GVAPLAHIAI YKVCNSVGCS ESDVLAAMDS AIDDGVDILS MSLGGGPIPF HRDNIAIGAY SATERGILVS
arcan NGHGTHTAST AAGAFVKGAN VYGNADGTAV GVAPLAHIAI YKVCNSVGCS ESDVLAAMDS AIDDGVDILS MSLGGGPIPF HRDNIAIGAY SATERGILVS
huayl NGHGTHTAST AAGAFVKGAN VYGNANGTAV GVAPLAHIAI YKVCNSVGCS ESDVLAAMDS AIDDGVDILS MSLGGGPIPF HRDNIAIGAY SATERGILVS
chile NGHGTHTAST AAGAFVKGAN VYGNANGTAV GVAPLSHIAI YKVCNSVGCS ESDVLAAMDS AIDDGVDILS MSLGGVPIPF HRDNIAIGAY SATERGILVS
penne NGHGTHTAST AAGAFVKGAN VYGNADGTAV GVAPLAHIAI YKVCNSVGCS ESDVLAAMDS AIDDGVDILS MSLGGGPIPF HRDNIAIGAY SATERGILVS
peruv NGHGTHTAST AAGAFVKGAN VYGNADGTAV GVAPLAHIAI YKVCNSVGCS ESDILAAMDS AIDDGVDILS MSLGGGPIPF HRDNIAIGAY SATERGILVS

      301                                                                                                      400
lycop CSAGNSGPSF ITAVNTAPWI LTVGASTLDR KIKATVKLGN GEEFEGESAY RPKISNATFF TLFDAAKNAK DPSETPYCRR GSLTDPAIRG KIVLCSALGH
pimpi CSAGNSGPSF ITAVNTAPWI LTVGASTLDR KIKATVKLGN GEEFEGESAY RPKISNATFF TLFDAAKNAK DPSETPYCRR GSLTDPAIRG KIVLCSALGH
chees CSAGNSGPSF ITAVNTAPWI LTVGASTLDR KIKATVKLGN GEEFEGESAY RPKISNATFF TLFDAAKNAK DPSETPYCRR GSLTDPAIRG KIVLCSALGH
habro CSAGNSGPSL ITAGNTAPWI LTVGASTLDR KIKATVKLGN GEEFEGESAY RPKISNSSFF TLYDAAKNAK DPSKTPYCRR GSLTDPAIRG KIVLCLALGH
corne CSAGNSGPSL VTAGNTAPWI LTVGASTLDR KIKATVKLGN GEEFEGESAY RPKISNATFF TLYDAAKNAK EQSKTPYCKP GSLTDPAIKG KIVLCLAFGD
neori CSAGNSGPSF ITAGNTAPWI LTVGASTLDR KIKATVKLGN GEEFEGESAY RPKISNSTFF TLFDAAKNAK DPSETPYCRR GSLTDPAIRG KIVLCSAFGG
arcan CSAGNSGPSF ITAGNTAPWI LTVGASTLDR KIKATVKLGN GEEFEGESAY RPKISNSTFF TLFDAAKNAK DPSETPYCRR GSLTDPAIRG KIVLCSAFGG
huayl CSAGNSGPSF ITAGNTAPWI LTVGASTLDR KIKATVKLGN GEEFEGESAY RPKISNSTFF TLFDAAKNAK DPSETPYCRR GSLTDPAIRG KIVLCSAFGR
chile CSAGNSGPSL VTAGNTAPWI LTVGASTLDR KIKATVKLGN GEEFEGESAY RPKISNATFF TLFDAAKNAK DPSETPYCRR GSLTDPAIRG KIVLCLAFGG
penne CSAGNSGPSL ITAGNTAPWI LTVGASTLDR KIKSTVKLGN GEEFEGESAY RPKISNSTFF TLFDAAKNAK DPSETPYCKP GSLTDPAIKG KIVLCLAFGG
peruv CSAGNSGPSL ITAGNTAPWI LTVGASTLDR KIRATVKLGN GEEFEGESAY RQKISNSTFF TLFDAAKNAK DPSETPYCRP GSLTDPAIRG KIVLCLAFGR

      401                                                                                                      500
lycop VANVDKGQAV KDAGGVGMII INPSQYGVTK SADAHVLPAL VVSAADGTKI LAYMNSTSSP VATIAFQGTI IGDKNAPMVA AFSSRGPSRA SPGILKPDII
pimpi VANVDKGQAV KDAGGVGMII INPSQYGVTK SADAHVLPAL VVSAADGTKI LAYMNSTSSP VATIAFQGTI IGDKNAPMVA AFSSRGPSRA SPGILKPDII
chees VAYVDKGQAV KDAGGVGMII INPSQYGVTK SADAHVLPAL VVSAADGTKI LAYMNSTSSP VATIAFQGTI IGDKNAPMVA AFSSRGPSRA SPGILKPDII
habro VANVDKGQAV KDAGGVGMIV INPSQYGVTK SADAHVLPAL DVSAADGTKI LAYMNSTSNP VATIAFQGTI IGDKNAPMVA AFSSRGPSRA SPGILKPDII
corne VANVDKGQAV KDAGGVGMII INPSQYGVTK SADAHVLPAL VVSAADGTKI LAYMNSTSSP VATIAFQGTI IGDKNAPMVA AFSSRGPSRV SPGILKPDII
neori VTNVDKGQAV KDAGGVGMII INPSQYGVTK SADAHVLPAL VVSAADGTKI LAYMNSTSSP VATIAFQGTI IGDKNAPMVA AFSSRGPSRA SPGILKPDII
arcan VTNVDKGQAV KDAGGVGMII INPSQYGVTK SADAHVLPAL VVSAADGTKI LAYMNSTSSP VATIAFQGTI IGDKNAPMVA AFSSRGPSRA SPGILKPDII
huayl VANVDKGQAV KDAGGVGMII INPSQYGVTK SADAHVLPAL VVSAADGTKI LAYMNSTSSP VATIAFQGTI IGDKNAPMVA AFSSRGPSRA SPGILKPDII
chile VANVDKGQAV KDAGGVGMII INPSQYGVTK SADAHVLPAL VVSAADGTKI LAYMNSTSSP VATIAFQGTI IGDKNAPMVA AFSSRGPSRA SPGILKPDII
penne VANVDKGQAV KDAGGVGMII INPSQYGVTK SADAHVLPAL VVSAADGTKI LAYMNSTSSP VATIAFQGTI IGDKNAPMVA AFSSRGPSRA SPGILKPDII
peruv VANVDKGQAV KDAGGVGMII INPSQYGVTK SADAHVLPAL VVSAADGTKI LAYMNSTSSP VATIAFQGTI IGDKNAPMVA AFSSRGPSRV SPGILKPDII

      501                                                                                                      600
lycop GPGANILAAW PTSVDDNKNT KSTFNIISGT SMSCPHLSGV AALLKCTHPD WSPAVIKSAM MTTADTLNLA NSPILDERLL PADIYAIGAG HVNPSRANDP
pimpi GPGANILAAW PTSVDDNKNT KSTFNIISGT SMSCPHLSGV AALLKCTHPD WSPAVIKSAM MTTADTLNLA NSPILDERLL PADIYAIGAG HVNPSRANDP
chees GPGANILAAW PTSVDDNKNT KSTFNIISGT SMSCPHLSGV AALLKCTHPD WSPAVIKSAM MTTADTLNLA NSPILDERLL PADIYAIGAG HVNPSRANDP
habro GPGANILAAW PTSVDDNKNT KSTFNIISGT SMSCPHLSGV AALLKCTHPD WSPAAIKSAM MTTADTLNLA NSPILDERLL PADIYAIGAG HVNPSRANDP
corne GPGANILAAW PTSVDDNKNT KSTFNIMYGT SMSCPHLSGV AALLKCTHPD WSPAAIKSAM MTTADTLNLA NSPILDERLL PADIYAIGAG HVNPSRANDP
neori GPGANILAAW PTSVDDNKNI KSTFNIMSGT SMSCPHLSGV AALLKCTHPD WSPAVIKSAM MTTADTLNLA NSPILDERLL PADIYAIGAG HVNPSRANDP
arcan GPGSNILAAW PTSVDDNKNT KSTFNIISGT SMSCPHLSGV AALLKCTHPD WSPAVIKSAM MTTADTLNLA NSPILDERLL PADIYAIGAG HVNPSRANDP
huayl GPGANILAAW PTSVDDNKNT KSTFNIISGT SMSCPHLSGV AALLKCTHPD WSPAVIKSAM MTTADTLNLA NSPILDERLL PADIYAIGAG HVNPSRANDP
chile GPGANILAAW PTSVDDNKNT KSTFNIMSGT SMSCPHLSGV AALLKCTHPD WSPAVIKSAM MTTADTLNLA NSPILDERLL PADIYAIGAG HVNPSRANDP
penne GSGANILAAW PTSVDDNKNT KSTFNIISGT SMSCPHLSGV AALLKCTHPD WSPAAIKSAM MTTADTLNLA NNPILDERLL PADIYAIGAG HVNPSRANDP
peruv GPGANILAAW PTSVDDNKNT KSTFNIISGT SMSCPHLSGV AALLKYTHPD WSPAAIKSAI MTTADTLNLA NSPILDERLL PADIYAIGAG HVNPSRANDP

      601                                                                                                      700
lycop GLVYDTPFED YVPYLCGLKY TDQQVGNLIQ RRVNCSEVKS ILEAQLNYPS FSIFGLGSTP QTYTRTVTNV GDATSSYKVE VASPEGVAIE VEPSELNFSE
pimpi GLVYDTPFED YVPYLCGLKY TDQQVGNLIQ RRVNCSEVKS ILEAQLNYPS FSIFGLGSTP QTYTRTVTNV GDATSSYKVE VASPEGVAIE VEPSELNFSE
chees GLVYDTPFED YVPYLCGLKY TDQQVGNLIQ RRVNCSEVKS ILEAQLNYPS FSIFGLGSTP QTYTRTVTNV GDATSSYKVE VASPEGVAIE VEPSELNFSE
habro GLVYDTPFED YLPYLCGLNY TNRQVGNLLQ RRVNCSEVKS ILEAQLNYPS FSIFGLGSTP QTYTRTVTNV GDATSSYKVE VASPKGVAIE VEPSELNFSE
corne GLVYDTPFED YVPYLCGLKY TDQQVGNLIQ RRVNCSEVKS ILEAQLNYPS FSIFGLGSTP QTYTRTVTNV GDATSSYKVE VASPKGVAVE VEPAELNFSE
neori GLVYDTPFED YVPYLCGLNY TNRQVGNLIQ RRVNCSEVKS ILEAQLNYPS FSIFGLGSTP QTYTRTVTNV GDATSSYKVE VASPKDVVVE VEPAELNFSE
arcan GLVYDTPFED YVPYLCGLNY TNRQVGNLIQ RRVNCSEVKS ILEAQLNYPS FSIFGLGSTP QTYTRTVTNV GDATSSYKVE VASPKEVVVE VEPAELNFSE
huayl GLVYDTPFED YVPYLCGLKY TDQQVGNLIQ RRVNCSEVKS ILEAQLNYPS FSIFGLGSTP QTYTRTVTNV GDATSSYKVE VASPKGVAVE VEPAELNFSE
chile GLVYDTPFED YVPYLCGLKY TDQQVGNLIQ RRVNCSEVKS ILEAQLNYPS FSIFGLGSTP QTYTRTVTNV GDAKSSYKVE VASPKDVVVE VEPAELNFSE
penne GLVYDTPSED YLPYLCGLKY TDQQVGNLLQ RKVNCSEVKS ILEAQLNYPS FSIFGLGSTP QTYTRTVTNV GDATSSYKVE VASPKDVVVE VEPAELNFSE
peruv GLVYDTPFED YVPYLCGLKY TDQQVGNLIQ RRVNCSEVKS ILEAQLNYPS FSIFGLGSTP QTYTRTVTNV GDATSSYKVE VASPKGVAIE VEPSELNFSE

      701                                       745
lycop LNQKLTYQVT FSKTTNSSNP EVIEGFLKWT SNRHSVRSPI AVVSA
pimpi LNQKLTYQVT FSKTTNSSNP EVIEGFLKWT SNRHSVRSPI AVVSA
chees LNQKLTYQVT FSKTTNSSNP EVIEGFLKWT SNRHSVRSPI AVVSA
habro LKQKLTYQVT FSKTSSSSNI EVVEGFLKWT SNRHSVRSPI AVVFA
corne LKQKLTYQVT FSKSSSSSNI EVVEGFLKWT SNRHSARSPI AVVFA
neori LKQKLTYQVT FSKSSSSSNI EVVEGFLKWT SSRHSVRSPI AVVFA
arcan LKQKLTYQVT FSKSSSSSNI EVVEGFLKWT SSRHSVRSPI AVVFA
huayl LKQKLTYQVT FSKSSSSSNI EVVEGFLKWT SNRHSVRSPI AVVFA
chile LKQKLTYQVT FSKSSSSSNI EVVEGFLKWT SNRHSVRSPI AVVLA
penne LKQKLTYQVT FSKTSSSSNI EVVEGFLKWT SNRHSVRSPI AVVFA
peruv LKQKLTYQVT FSKSSSSSNI EVVEGFLKWT SNRHSVRSPI AVVFA
```

Supplementary Figure 3 Amino acid alignment of P69B orthologs from wild tomato species. P69B orthologs were extracted from genomes from wild tomato species and aligned. Residues that are different from P69B of cultivated tomato are highlighted in light grey, dark grey or red for n = 2, 3 and 4 variant residues, respectively. Note that only position 400 has 4 different residues.

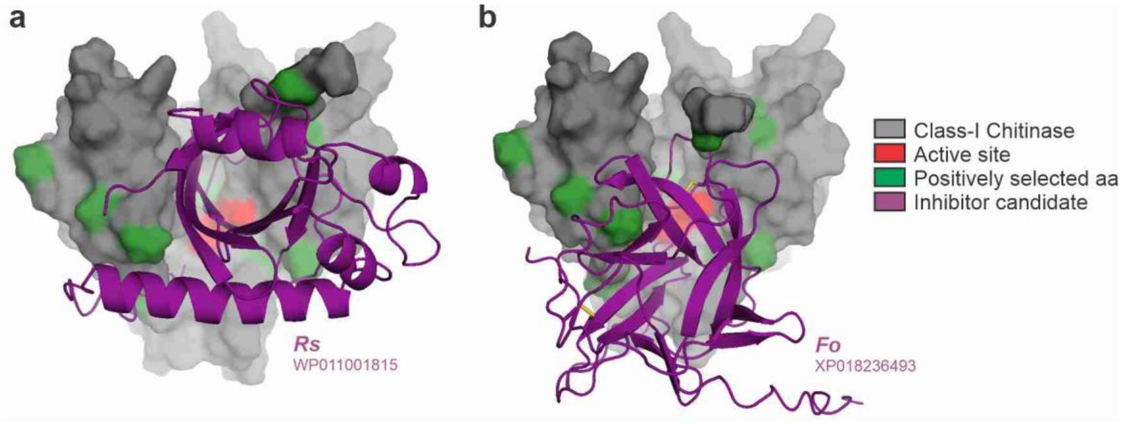

Supplementary Figure 4 Two candidate inhibitors interact with positively selected sites in Class-I chitinases.

Positively selected sites reported previously (Bishop et al., 2000) were plotted onto the AFM-predicted models of tomato Class-I chitinase in complex with inhibitors from *Ralstonia solanacearum* **(a)** and *Fusarium oxysporum f. sp. lycopersici* **(b)**.

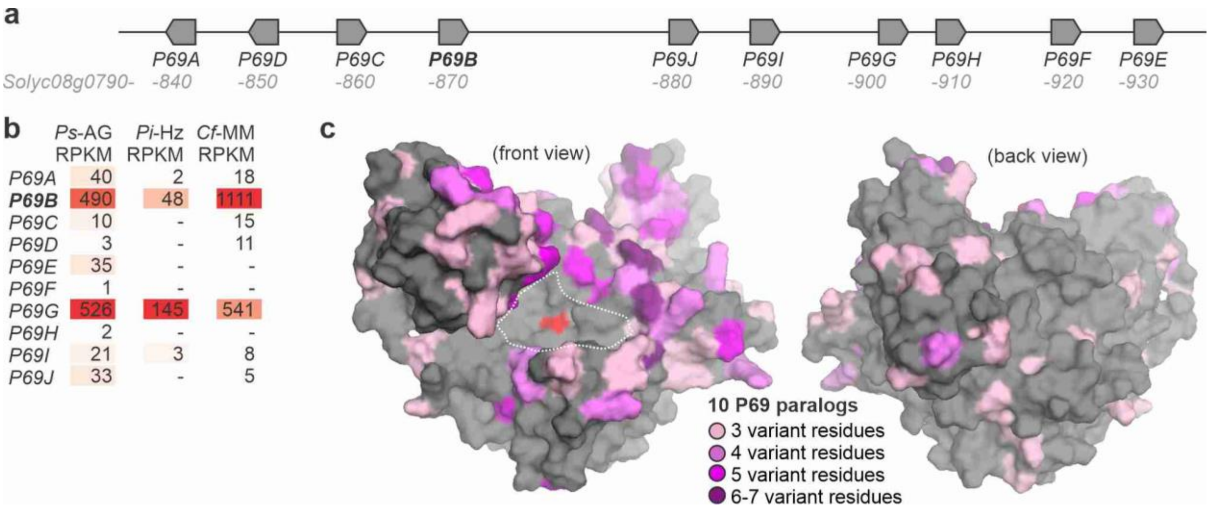

Supplementary Figure 5 Expression and variation of the ten P69 paralogs of tomato.

a Genomic P69 cluster contains ten paralogs. Sequence was extracted from the genome of cv. Heinz (Tomato Genome Consortium, 2012). **b** Expression levels in reads per kilobase per million (RPKM) of *P69* genes in three different cultivars infected with *Ps* (cv. Ailsa Graig, Rosli et al., 2013); *Pi* (cv. Heinz, Hwang et al., 2020) and *Cf* (cv. Money Maker, Ilyas et al., 2016). **c** Protein sequences of the ten P69 paralogs of tomato (P60A-I) were aligned and the number of variant residues at each position was plotted on the model of the P69B structure. The variation (purple) is surrounding the active site (red) in the substrate binding groove (dashed line) as a ‘ring-of-fire’.

Supplementary Table 1 RMSD and TM scores for control complexes.

AFM model	Structure	Comparison of monomers	RMSD (Å)	TM
Pip1-EpiC2B (compatible)	3IMA	Pip1 - Papain	**0.94**	**0.9517**
		EpiC2B - Tarocystatin	2.37	**0.7827**
		Interface*	**0.85**	**0.89**
P69B-Epi1a (compatible)	1YU6	P69B - Subtilisin	**1.74**	**0.9217**
		Epi1a - OMTKY3	**1.44**	0.5541
		Interface*	**1.12**	**0.83**
Pip1-Epi1a (incompatible)	3IMA	Pip1 - Papain	**0.96**	**0.9509**
	1YU6	Epi1a - OMTKY3	**1.72**	0.5252
P69B-EpiC2B (incompatible)	1YU6	P69B - Subtilisin	**1.77**	**0.9220**
	3IMA	EpiC2B - Tarocystatin	2.47	**0.7687**

*, Calculated from the PDB files by identifying residues that have differential solvent exposure when the complex partner is omitted.

DALI was used to identify experimentally-resolved structures with similarity to AFM-predicted control complexes: tarocystatin-papain (3IMA) for Pip1-EpiC2B and subtilisin-OMTKY3 (1YU6) for P69B-Epi1a. TMalign was used to calculate TM and RMSD values for the monomers in the AFM model compared to the resolved structures. The interface was determined by selecting the residues that show different solvent exposure when the complex partner is omitted. Scores that are above the set threshold are printed in green bold and below the threshold in red.

Supplementary Table 2 Analysed RNAseq samples

SRA run ID	Pathogen	Tomato cultivar	Tissue/time
SRR5467166	*Rs* GMI1000 WT	'Bonny Best'	Petiole/3dpi
SRR5467167	*Rs* GMI1000 WT	'Bonny Best'	Petiole/3dpi
SRR5467168	*Rs* GMI1000 WT	'Bonny Best'	Petiole/3dpi
SRR6924534	*Bc* B05.10 WT	'Marmande'	Leaf/1dpi
SRR6924535	*Bc* B05.10 WT	'Marmande'	Leaf/1dpi
SRR6924536	*Bc* B05.10 WT	'Marmande'	Leaf/1dpi
SRR6050413	*Fol* 4287 WT	'Moneymaker' (S)	Root/1dpi
SRR6050414	*Fol* 4287 WT	'Motelle' (R)	Root/1dpi
SRR1171035	*Cf* race-0WU	'Heinz Cf-0' (S)	Leaf/4dpi
SRR1171040	*Cf* race-0WU	'Heinz Cf-0' (S)	Leaf/8dpi
SRR1171043	*Cf* race-0WU	'Heinz Cf-0' (S)	Leaf/12dpi
SRR1171047	*Cf* IPO1979	'Moneymaker'	Leaf/6dpi

Supplementary Table 3 Transcript levels of six tomato genes in (non)infected plants

Cladosporium fulvum		Day-6, susceptible tomato (n=3) Mean±SD		Day-6, resistant tomato (n=3) Mean±SD	
name	Accession	Mock	*Cf*	Mock	Cf
P69B	Solyc08g079870.3.1	352 ± 371	1111 ± 283	221 ± 56	2628 ± 117
Pip1	Solyc02g077040.4.1	223 ± 263	661±304	118 ± 34	1358 ± 166
A1P	Solyc08g067100.2.1	78 ± 8	88 ± 4	72 ± 9	116 ± 10
C-I	Solyc09g098540.3.1	234 ± 14	195 ± 7	224 ± 12	168 ± 13
C-III	Solyc05g050130.4.1	90 ± 103	232 ± 65	44 ± 18	377 ± 35
C-V	Solyc07g005090.4.1	28 ± 9	51 ± 9	24 ± 7	61 ± 6
Fusarium oxysporum		Day 1, susceptible tomato (n=1)		Day 1, resistant tomato (n=1)	
name	Accession	Mock	*Fo*	Mock	*Fo*
P69B	Solyc08g079870.1.1	3.69	17.09	1.49	5.00
Pip1	Solyc02g077040.2.1	2.14	6.49	3.21	3.62
A1P	Solyc08g067100.2.1	290.53	339.77	285.60	347.14
C-I	Solyc09g098540.2.1	446.14	316.62	363.06	311.31
C-III	Solyc05g050130.2.1	61.33	141.36	60.73	89.85
C-V	Solyc07g005090.2.1	172.49	210.57	168.12	172.49
Phytophthora infestans		Days 1/2/3, susceptible tomato, Pi (n=4) Mean±SD			
name	Accession	Mock	1dpi *Pi*	2dpi *Pi*	3dpi *Pi*
P69B	Solyc08g079870.1.1	37 ± 1	107 ± 3	48 ± 2	46 ± 1
Pip1	Solyc02g077040.2.1	39 ± 1	24 ± 1	19 ± 1	16 ± 1
A1P	Solyc08g067100.2.1	48 ± 1	60 ± 1	40 ± 1	28 ± 1
C-I	Solyc09g098540.2.1	140 ± 3	158 ± 5	76 ± 1	41 ± 1
C-III	Solyc05g050130.2.1	13 ± 1	131 ± 5	92 ± 2	43 ± 2
C-V	Solyc07g005090.2.1	10 ± 1	28 ± 1	32 ± 1	59 ± 1
Botrytis cinerea		Day 1, susceptible tomato, WT *Bc* (n=3)			
name	Accession	Rep1	Rep2	Rep3	Mean±SD
P69B	Solyc08g079870.1.1	5.77	2.74	10.18	6 ± 4
A1P	Solyc08g067100.2.1	19.80	11.35	20.89	17 ± 5
C-I	Solyc09g098540.2.1	10.95	4.75	17.27	11 ± 6
C-III	Solyc05g050130.2.1	0	0	0	0
C-V	Solyc07g005090.2.1	265.26	286.11	451.3	334 ± 102
Ralstonia solanacearum		Day-3, susceptible tomato, *Rs* (n=3)			
name	Accession	Rep1	Rep2	Rep3	Mean±SD
P69B	Solyc08g079870.1.1	503.40	954.43	734.40	731±226
A1P	Solyc08g067100.2.1	97.34	101.76	89.39	96±6
C-I	Solyc09g098540.2.1	905.04	867.27	871.69	881±21
C-III	Solyc05g050130.2.1	661.37	1111.08	865.27	879±225
C-V	Solyc07g005090.2.1	54.25	56.65	45.60	52±6

FPKM values of tomato genes, extracted from Ilyas et al., 2016 (*Cf*); Zhao et al., 2018 (*Fo*); Huang et al., 2020 (*Pi*); Müller et al., 2018 (*Bc*); Khokhani et al., 2017 (*Rs*).

Supplementary Table 4 RNA-seq values of SSPs in infected plants*

Cf	4dpi SRR1171035	6dpi SRR1171047	8dpi SRR1171040	12dpi SRR1171043	Mean(±SD)
KAH3648627 (*Cf*Ecp36)	212.485	436.287	578.257	691.188	479.55 ± 206.34
Fo	MoneyMaker (S) SRR6050413	Motelle (R) SRR6050414	Mean(±SD)		
XP018243121 (*Fo*TIL)	180	461	340.50 ± 198.70		
APP91304 (*Fo*Six15)	270.034	144.81	207.42 ± 88.55		
XP018236493	204.966	227.051	216.01 ± 15.62		
XP018248187	5.39765	5.71688	5.56 ± 0.23		
XP018241286	1.79816	5.8976	3.85 ± 2.90		
Rs	SRR5467166	SRR5467167	SRR5467168	Mean(±SD)	
WP011000405	0	6.374	13.972	6.78 ± 6.99	
WP011001815	528.431	656.721	612.85	599.33 ± 65.20	
WP011002292	2.7102	2.8454	3.11861	2.89 ± 0.21	
Bc	SRR6924534	SRR6924535	SRR6924536	Mean(±SD)	
XP001545484	2.47535	2.34842	2.50272	2.44 ± 0.82	
XP001560184	71.8476	79.0248	27.7998	59.56 ± 27.7	

*, all values are in transcripts per million (TPM) reads of the pathogen during infection of tomato. Infection of *Rs* and *Bc* was at 72 and 24 hpi, respectively.

Supplementary Table 5 RMSD and TM scores for the hydrolases in 15 SPP-hydrolase complexes

Hydrolase	SSP	PDB	Protein(s) in PDB	RMSD (Å)	TM
P69B	WP046932418-*Xp*Ssp	1YU6	Subtilisin-OMTKY3	**1.7**	**0.92**
P69B	KAH3648627-*Cf*Ecp36	1YU6	Subtilisin-OMTKY3	**1.7**	**0.92**
P69B	XP018243121-*Fo*TIL	1YU6	Subtilisin-OMTKY3	**1.7**	**0.92**
P69B	APP91304-*Fo*Six15	1YU6	Subtilisin-OMTKY3	**1.7**	**0.92**
P69B	XP001545484	1YU6	Subtilisin-OMTKY3	**1.7**	**0.92**
P69B	WP011000405	1YU6	Subtilisin-OMTKY3	**1.8**	**0.92**
P69B	WP008576433	1YU6	Subtilisin-OMTKY3	**1.8**	**0.92**
C-I	WP011001815	3IWR	Rice class-I chitinase	**1.4**	**0.95**
C-I	XP018236493	3IWR	Rice class-I chitinase	**1.4**	**0.92**
C-III	WP046931881	4TOQ	Class-III chitinase	**0.75**	**0.97**
C-III	XP001560184	4TOQ	Class-III chitinase	**0.76**	**0.97**
C-III	XP018248187	4TOQ	Class-III chitinase	**0.79**	**0.97**
C-III	XP018241286	4TOQ	Class-III chitinase	**0.86**	**0.97**
C-V	WP008572913	3ALG	Class-V chitinase	**1.4**	**0.94**
A1P	WP011002292	4ZL4	Plasmepsin	3.0	0.73

DALI was used to identify the experimentally resolved structure that is closest to that of the hydrolase in the SPP-hydrolase complex and the RMSD and TM scores were calculated for each comparison with TMalign. Scores that are above the set threshold are printed in green bold and below the threshold in red.

Supplementary Table 6 RMSD and TM scores for the SSPs in 15 SPP-hydrolase complexes

SSP	PDB	RMSD (Å)	TM
WP046932418-*Xp*Ssp	x	x	x
KAH3648627-*Cf*Ecp36	6NK9	**1.9**	0.63
XP018243121-*Fo*TIL	x	x	x
APP91304-*Fo*Six15	x	x	x
XP001545484	1WPX	2.6	0.68
WP011000405	x	x	x
WP008576433	x	x	x
WP011001815	5UC0	4.7	0.53
XP018236493	4I1B	3.6	0.62
WP046931881	4MIS	2.2	0.70
XP001560184	3TC2	3.7	0.61
XP018248187	5FJQ	2.7	0.46
XP018241286	4OWL	3.0	0.70
WP008572913	1Y34	2.8	0.66
WP011002292	2HOD	3.0	0.61

DALI was used to identify the resolved structure that is closest to that of the SSP in the SPP-hydrolase complex and the RMSD and TM scores were calculated for each comparison with TMalign. Scores that are above the set threshold are printed in green bold and below the threshold in red. No good structures were identified for several complexes (x).

Supplementary Table 7 Synthesised nucleotide sequences

P69B Fragment-1
GAAGACATAATGGGATTTGTTCTCTTTTCACAATTGCCTTCATTTCTTCTTGTCTCTACACTTCTCTTATTCCT
AGTAATATCCCACTCTTGCCGTGCAGGAGGTAATTTAGAGACTTACATCGTCCACGTGGAGAGTCCAGAAT
CTTTGGTCACCACTCAATCACTCTTGACAGATTTGGGTAGTTACTATCTTTCCTTTCTGCCCAAAACGGCTAC
TACGATCTCCTCAAGTGGCAACGAGGAAGCCGCGACAATGATTTACTCATATCATAACGTAATGACTGGTT
TCGCAGCTAGATTAACTGCTGAGCAGGTCAAAGAAATGGAAAAGAAACATGGATTTGTGTCCGCACAGAA
GCAAAGAATCTTGAGCCTACATACTACACACACTCCTAGTTTCCTTGGTTTACAGCAAAATATGGGCGTTTG
GAAGGACTCAAATTATGGTAAGGGAGTGATCATTGGTGTAATTGATACAGGTATCATTCCCGACCATCCCA
GCTTTAGTGATGTTGGCATGCCCCCGCCACCTGCAAAATGGAAAGGTGTTTGCGAGTCTAATTTTACTAATA
AGTGTAATAACAAGCTTATAGGTGCCCGATCTTACCAACTAGGTAATGGTTCCCCAATTGATTCTATAGGGC
ACGGAACGCACACAGCCAGTACTGCTGCCGGCGCTTTCGTAAAAGGAGCCAACGTGTATGGTAATGCCGAC
GGAACAGCCGTAGGGGTGGCACCATTAGCTCATATTGCTATTTATAAGGTCTGCAATTCAGTTGGTTGTTCA
GAGTCAGACGTACTTGCAGCCATGGACTCCGCAATAGACGATGGTGTTGATATACTTTCTATGTCTTTGTCT
GGAGGTCCGATTCCTTTTCACCGGGACAATATCGCTATAGGGGCCTATAGTGCTACTGAACGTGGAATCCTC
GTTTCTTGTTCAGCTGGTAATAGTGGACCTTCCTTTATTACAGCTGTCAATACCGCTCCTTGGATTCTAACTG
TCGGAGCTTCCACTTTGGACCGTAAGATTAAGGCAACTGTGAAACTGGGCAACGGTGAGGAATTTGAAGGA
GAATCAGCATATCGTCCTAAGATAAGCAATGCTACATTCTTTACACTCTTTGATGCGGCAAAGAACGCTAA
AGACCCTAGCGAAACCCCCTATTGTCGCAGGGGAAGTCTAACAGACCCTGCTATCAGGGGTAAAATAGTTC
TCTGCAGTGCTCTGGGACATGTTGCAAACGTTGATAAAGGACAGGCGGTAAAGGACGCTGGTGGAGTTGGA
ATGATAATCATAAATCCAAGTCAGTATGGAGTAACCAAGTCTGCTGATGCTCATGTGTTGCCTGCTCTCGTA
GTGAGCGCCGCTGATGGTATGTCTTC

P69B Fragment-2
GAAGACATTGGTACTAAGATATTGGCATATATGAACTCTACTAGCTCTCCAGTAGCCACAATTGCATTTCAG
GGAACGATCATTGGTGACAAGAACGCACCGATGGTTGCGGCATTTAGTTCCCGAGGCCCTTCACGTGCATC
CCCTGGTATCCTCAAGCCTGATATCATTGGACCTGGCGCAAACATACTTGCCGCATGGCCTACTTCAGTGGA
TGACAATAAGAACACAAAGTCTACTTTTAATATAATTAGTGGTACAAGCATGTCCTGTCCCCACCTGTCTGG
AGTAGCTGCACTGCTTAAATGTACGCATCCAGATTGGTCACCAGCTGTTATAAAGAGCGCTATGATGACGA
CCGCTGACACCTTGAACCTCGCAAACTCTCCTATTCTTGACGAGCGTTTGCTTCCAGCTGACATCTATGCAA
TAGGCGCCGGACATGTGAATCCGAGTAGGGCTAATGACCCAGGGTTGGTGTACGATACTCCTTTCGAAGAT
TACGTCCCATATCTCTGTGGACTTAAATATACGGATCAACAGGTTGGTAATTTAATTCAAAGACGTGTAAAC
TGTTCCGAAGTTAAGAGCATCTTGGAAGCTCAACTGAATTATCCCAGTTTTTCTATTTTTGGACTAGGAAGT
ACTCCTCAGACTTATACAAGAACTGTGACTAATGTCGGAGATGCAACATCTTCCTACAAAGTTGAGGTTGC
CAGCCCTGAAGGGGTCGCAATCGAAGTTGAACCTAGCGAGCTTAACTTTTCCGAGCTAAATCAGAAACTTA
CGTACCAAGTAACATTTTCAAAGACAACTAACTCTAGTAATCCAGAAGTAATCGAGGGTTTTCTTAAATGG
ACTAGTAATCGTCATAGTGTGCGATCTCCCATCGCCGTTGTAAGCGCAGGTGGGCACCATCACCATCACCA
CTGAGCTTATGTCTTC

His-MBP-TEV
ATGGGCAGCAGCCATCATCATCATCATCATATGAAAATCGAAGAAGGTAAACTGGTAATCTGGATTAACGG
CGATAAAGGCTATAACGGTCTCGCTGAAGTCGGTAAGAAATTCGAGAAAGATACCGGAATTAAAGTCACC
GTTGAGCATCCGGATAAACTGGAAGAGAAATTCCCACAGGTTGCGGCAACTGGCGATGGCCCTGACATTAT
CTTCTGGGCACACGACCGCTTTGGTGGCTACGCTCAATCTGGCCTGTTGGCTGAAATCACCCCGGACAAAG
CGTTCCAGGACAAGCTGTATCCGTTTACCTGGGATGCCGTACGTTACAACGGCAAGCTGATTGCTTACCCGA
TCGCTGTTGAAGCGTTATCGCTGATTTATAACAAAGATCTGCTGCCGAACCCGCCAAAAACCTGGGAAGAG
ATCCCGGCGCTGGATAAAGAACTGAAAGCGAAAGGTAAGAGCGCGCTGATGTTCAACCTGCAAGAACCGT
ACTTCACCTGGCCGCTGATTGCTGCTGACGGGGGTTATGCGTTCAAGTATGAAAACGGCAAGTACGACATT
AAAGACGTGGGCGTGGATAACGCTGGCGCGAAAGCGGGTCTGACCTTCCTGGTTGACCTGATTAAAAACAA
ACACATGAATGCAGACACCGATTACTCCATCGCAGAAGCTGCCTTTAATAAAGGCGAAACAGCGATGACC
ATCAACGGCCCGTGGGCATGGTCCAACATCGACACCAGCAAAGTGAATTATGGTGTAACGGTACTGCCGAC
CTTCAAGGGTCAACCATCCAAACCGTTCGTTGGCGTGCTGAGCGCAGGTATTAACGCCGCCAGTCCGAACA
AAGAGCTGGCAAAAGAGTTCCTCGAAAACTATCTGCTGACTGATGAAGGTCTGGAAGCGGTTAATAAAGA
CAAACCGCTGGGTGCCGTAGCGCTGAAGTCTTACGAGGAAGAGTTGGCGAAAGATCCACGTATTGCCGCCA
CTATGGAAAACGCCCAGAAAGGTGAAATCATGCCGAACATCCCGCAGATGTCCGCTTTCTGGTATGCCGTG
CGTACTGCGGTGATCAACGCCGCCAGCGGTCGTCAGACTGTCGATGAAGCCCTGAAAGACGCGCAGACTA
ATTCGAGCTCGAACAACAACAACAATAACAATAACAACAACCTCGGGATCGAGGAAAACCTGTATTTTCAG
GGCGAATTCGGATCCTCTAGAGTCGACCTGCAGGCAAGCTT

***Xp*Ssp1-WP046932418.1-P3**
GCTCCACCTACAGATACGGCAACTCCCCCCCCAGCGACGGGTTCCACCCGTGGTCCGGCGGCTGGCAAGGC
GGTCGTGGTGACCACCACGTGCCGTACCGACGCCGACTGTACCGTGAAGAACGTAGGTAATTGTTGTGGCG
CATTTCCGGCGTGCGTGAACGTCAACAGCGCGACCGACCCGAATGGTGTTTTGGCTCAGTGCCAAGCGGGT
GGCATGATGAGCGTTTGCGGCTTCCGCGAGATCTCTGCCTGCCAGTGCGTTGCAGGCCAGTGCGCAGCTAA
AGATGCCCAAGCGGATACCCTGCGTCCGGCGACTCCGCCGACGGAAACCGTTCACTAG

***Cf*Ecp36-KAH3648627.1-P5**
AAAAGGGGGTCAGGTGGAGAATGTAGAACACAATCCGACTGTTGTTACTGCGACCCGGGTTCCGGCTGCAT
CCTGAGCTGTCAGGCAGATCGTTCTAGCACCACGGGCTATCGCTGCATTACCAGCCGTCAGTCGGCTTGCA
ACGTTGCGGGTTGCAGCTGCTAG

***Fo*TIL-XP_018243121.1-P8**<br>GCTCCCAAGTGTAAAGCAGGAGAACAATTTTTCGAGTGCGGCACCGCTTGCCCGTTGACGTGCGACAAACC<br>GGAACCGCGTCCGTGTACCAAACAATGTGTTCCGGGTTGCTTTTGCAAGGAGGTGCGCATTCTGCGTCAAA<br>GCCAGCAGGTTCTGTCGCCGCTCAAGGCGCTGTTGCTGCCGGTGAGCATCCACAACCGCCCAATCTCTGGT<br>ATTTCCCATAGCCGTCACCTGTAG
***Fo*Six15-APP91304.1-P9**<br>ACAATATATTGTAGGGATGTATCACCACCCCGCGATACCCGTAGCTGGTGTAAAACGAACACCCCGGCGTG<br>GCAAGGTTGCCAGCGTTTTTGCAGCGAGCACTGCCGCTCCACCCCGCGTGACTACCCGGACGGCTGCATGT<br>ATCATCTGCAGGTTGGCGGTGATTACGACTGTTTCTGCAAGTAG
**XP001545484.1-P6**<br>TTCACACCACCCGGATTTGAACCTAGTTCAACGAACGATCTGACCGTCGCATACGGCTCGAAACTTGCCAC<br>CAACGGTATTCAAATGCTGCGTGCGGACACCGCGTATGCTCCGATTCTGGGTACAGCGACCAAAATGTCCG<br>GCACCTATGCGGTTATGATGGTTGATCCGGACATCCCGCCGGCAAAGGTGGGTGGTGACACCAGCCAATTT<br>CTGCATTGGATGCAGGCAGACTTGACCAGCTCAAATACTACTACGACCATTGGTGGCCAGAAAATCTACGA<br>GCTGATTAACGTGAAGAATACCTCTGCCTTTGCGACGTACCTGCAGCCGAATCCGCCAGATATCGCTCCGA<br>CCACCCACCGTTATACCCAGTTGCTCTTTAACACCACTGGCATGAATATGAGCTTGGCCAGCCTGCAAACG<br>GCGGGTAAAACCCGCGGTAATTTCAACGCGACGAACGTTGTTAAGTCTGCTAAGTTAACGGTGCTGATGGG<br>TAACTCCTTCGACGTGAGCTTCGGCGATAAAGCGATCAACAACACCGGCGCGTCCACCGGCAGCAGCACCT<br>AG
**WP_011000405.1-P7**<br>GCTGATGATGTTCCCGTAGCGGACGCATCACTCCAACCGAGCGTTGAGGCGGCGCGCCCACCGGTTGCTGC<br>GGCGACCGTCGTGCAAGGTTTTGGCCAGGCAGTTGAGGCAAGCGCACTGGAAGGTTATCGTGGTGGTACGC<br>AGCCGCTGTACCAGACCGTCAACGACGCGCGTTTGTCCGGCACGGTGACCGATAATGCCGCGATTAACGTT<br>GCTACTGGCGCTAATATTGTACGTGACGGCAGCTTCGCCAACGCCTCTGGCATCCCGACCGTTATTCAAAAC<br>ACCGGTGCGAACGTGCTGATCCAGAATGCGACCATCGTGAACGTGCAGTTCCGCCCGTAG
**WP008576433.1-P4**<br>CTGTATTTTCAGGGCGAATTCGGTTGTAATACAGTAGCTGGAGCAGGGAAAGACATGCAGGGTGCGGGTGA<br>TAAAGTTGAGAAGACCGCTGAAAAGTGCAGCGACGGCAAATGTTAGGAATTCGGATCCTCTAGAGTC

Supplementary Table 8 Used plasmids

Plasmid	Description	Reference
pJK187	Binary vector (pL0V2-2x35S::SC)	This work
pET-32/28	Bacterial expression vector	Novinec et al., 2012
P19	Binary vector carrying p19 silencing inhibitor	Van der Hoorn et al., 2003
pFH20	Binary vector for P69B-His expression	This work
pHJ000	Bacterial expression vector pET-32/28-His-MBP-TEV	This work
pHJ028	Bacterial expression P3(WP046932418.1, *Xp*Ssp1)	This work
pHJ033	Bacterial expression P5(KAH3648627.1, *Cf*Ecp36)	This work
pHJ029	Bacterial expression P6(XP001545484.1,)	This work
pHJ032	Bacterial expression P7(WP011000405.1)	This work
pHJ030	Bacterial expression P8(XP_018243121.1, *Fo*TIL)	This work
pHJ031	Bacterial expression P9(APP91304.1, *Fo*Six15)	This work
pHJ043	Bacterial expression P4(WP008576433.1)	This work
pHJ046	Bacterial expression *Pi*Epi1	This work
pHJ047	Bacterial expression *Pi*EpiC1	This work

Supplementary Table 9 Primer sequences

Primer	Use	Nucleotide sequence (5'-3')
His-MBP-F	Cloning	AAGAAGGAGATATACCATGGGCAGCAGCCATCATCATCAT CATCATATG
His-MBP-R	Cloning	TGGTGGTGGTGGTGCTCGAGAAGCTTGCCTGCAG
Epi1-F	Cloning	CTGTATTTTCAGGGCGAATTCCAAAGCCCGCAAGTCATCAG
Epi1-R	Cloning	GACTCTAGAGGATCCGAATTCTTATCCCTCCTGCGGTGTC
EpiC1-F	Cloning	CTGTATTTTCAGGGCGAATTCCAAGTGGACGGCGGATACTC
EpiC1-R	Cloning	GACTCTAGAGGATCCGAATTCCTACTTAACTGGGGTAATCG
P3-F	Cloning	CTGTATTTTCAGGGCGAATTCGCTCCACCTACAGATACGG
P3-R	Cloning	GACTCTAGAGGATCCGAATTCCTAGTGAACGGTTTCCGTCGG
P4-F	Cloning	CTGTATTTTCAGGGCGAATTC
P4-R	Cloning	GACTCTAGAGGATCCGAATTC
P5-F	Cloning	CTGTATTTTCAGGGCGAATTCAAAAGGGGGTCAGGTGGAGA
P5-R	Cloning	GACTCTAGAGGATCCGAATTCCTAGCAGCTGCAACCCGCAA
P6-F	Cloning	CTGTATTTTCAGGGCGAATTCTTCACACCACCCGGATTTGA
P6-R	Cloning	GACTCTAGAGGATCCGAATTCCTAGGTGCTGCTGCCGGTGG
P7-F	Cloning	CTGTATTTTCAGGGCGAATTCGCTGATGATGTTCCCGTAGC
P7-R	Cloning	GACTCTAGAGGATCCGAATTCCTACGGGCGGAACTGCACGT
P8-F	Cloning	CTGTATTTTCAGGGCGAATTCGCTCCCAAGTGTAAAGCAGG
P8-R	Cloning	GACTCTAGAGGATCCGAATTCCTACAGGTGACGGCTATGGG
P9-F	Cloning	CTGTATTTTCAGGGCGAATTCACAATATATTGTAGGGATGT
P9-R	Cloning	GACTCTAGAGGATCCGAATTCCTACTTGCAGAAACAGTCGT
PJK187-F	Sequencing	CTATCCTTCGCAAGACCCTTC
PJK187-R	Sequencing	CTCAACACATGAGCGAAACC
pet32a-F	Sequencing	TAATACGACTCACTATAGGG
pet32a-R	Sequencing	GCTAGTTATTGCTCAGCGG
