# Supplementary material for: AlphaFold-Multimer predicts cross-kingdom interactions at the plant-pathogen interface
Source: Nat Commun. 2023 Sep 27;14:6040. doi: 10.1038/s41467-023-41721-9 (PMC10533508; doi:10.1038/s41467-023-41721-9)
Supplement: Supplementary file 3 — Description of Additional Supplementary Files [file 41467_2023_41721_MOESM3_ESM.pdf]

Description of Additional Supplementary Files:

Supplementary Data 1: Used scripts (.zip file)

Supplementary Data 2: Protein sequences of inhibitors and proteases used for Fig.1 (.doc)

Supplementary Data 3: PDB files of 5 controls and 15 candidate complexes (.zip)

Supplementary Data 4: Sequences of six hydrolases used for AFM screen (.doc)

Supplementary Data 5: Sequences of mature SSPs used for AFM screen (.fasta)

Supplementary Data 6: Raw values for graphs (.xlsx)

Supplementary Data 7: All ipTM+pTM scores for 11,274 protein pairs (.tsv)

Supplementary Data 8: Best ipTM+pTM scores for 11,274 protein pairs (.tsv)
