# Supplementary material for: AlphaFold-Multimer predicts cross-kingdom interactions at the plant-pathogen interface
Source: Nat Commun. 2023 Sep 27;14:6040. doi: 10.1038/s41467-023-41721-9 (PMC10533508; doi:10.1038/s41467-023-41721-9)
Supplement: Supplementary file 5 — Supplementary Data 2 [file 41467_2023_41721_MOESM5_ESM.docx]

**Supplemental File S2** Protein sequences of inhibitors and proteases used for Figure 1

>P69B *Solanum lycopersicum* Solyc08g079870.3.1 CAA76725.1

MGLLKILLVFIFCSFQWPTIQSNLETYIVHVESPESLVTTQSLLTDLGSYYLSFLPKTATTISSSGNEEAATMIYSYHNVMTGFAARLTAEQVKEMEKKHGFVSAQKQRILSLHTTHTPSFLGLQQNMGVWKDSNYGKGVIIGVIDTGIIPDHPSFSDVGMPPPPAKWKGVCESNFTNKCNNKLIGARSYQLGNGSPIDSIGHGTHTASTAAGAFVKGANVYGNADGTAVGVAPLAHIAIYKVCNSVGCSESDVLAAMDSAIDDGVDILSMSLSGGPIPFHRDNIAIGAYSATERGILVSCSAGNSGPSFITAVNTAPWILTVGASTLDRKIKATVKLGNGEEFEGESAYRPKISNATFFTLFDAAKNAKDPSETPYCRRGSLTDPAIRGKIVLCSALGHVANVDKGQAVKDAGGVGMIIINPSQYGVTKSADAHVLPALVVSAADGTKILAYMNSTSSPVATIAFQGTIIGDKNAPMVAAFSSRGPSRASPGILKPDIIGPGANILAAWPTSVDDNKNTKSTFNIISGTSMSCPHLSGVAALLKCTHPDWSPAVIKSAMMTTADTLNLANSPILDERLLPADIYAIGAGHVNPSRANDPGLVYDTPFEDYVPYLCGLKYTDQQVGNLIQRRVNCSEVKSILEAQLNYPSFSIFGLGSTPQTYTRTVTNVGDATSSYKVEVASPEGVAIEVEPSELNFSELNQKLTYQVTFSKTTNSSNPEVIEGFLKWTSNRHSVRSPIAVVSA

>Pip1 *Solanum lycopersicum* Solyc02g077040.4.1

MASNFFLKNITVVLLLFSILSLYPFIVTSRNLKELSMLERHENWMVHHGRVYKDDIEKEHRFKTFKENVEFIESFNKNGTQRYKLAINKYADLTTEEFTTSFMGLDTSLLSQQESTATTTSFKYDSVTEVPNSMDWRKRGSVTGVKDQGVCGCCWAFSAAAAIEGAYQIANNELISLSEQQLLDCSTQNKGCEGGLMTVAYDFLLQNNGGGITTETNYPYEEAQNVCKTEQPAAVTINGYEVVPSDESSLLKAVVNQPISVGIAANDEFHMYGSGIYDGSCNSRLNHAVTVIGYGTSEEDGTKYWIVKNSWGSDWGEEGYMRIARDVGVDGGHCGIAKVASFPTA

>Epi1 *Phytophthora infestans* PITG_22681

MKSALLFTLVVAAVHAQSPQVISPAPRRESNEIDCPEYCLDVYDPVGDGEGNTYSNECYMKRAKCHNETTPPAWKDLVLITGSSTGEQPPSKKCSTVCPDVELPVCGSNRVRYGNPCELRIAACEHPELNIVEDSGKACVGSKVTPQEG

>EpiC2B *Phytophthora infestans* PITG_09173

MSFLRPTLALLAVTALVTTSAQLNGYSKKEVTPEDTELLQKAQSNVSAYNSDVTSRICYLKVDSLETQVVSGENYKFHVSGCSVNSDKELGGCANQNCESSKYDIVIYSQSWTNTLKVTSITPAN

Highlighted are the predicted signal peptide (grey) and the mature proteins used for AFM modeling (underlined)
