# Supplementary material for: AlphaFold-Multimer predicts cross-kingdom interactions at the plant-pathogen interface
Source: Nat Commun. 2023 Sep 27;14:6040. doi: 10.1038/s41467-023-41721-9 (PMC10533508; doi:10.1038/s41467-023-41721-9)
Supplement: Supplementary file 12 — Reporting Summary [file 41467_2023_41721_MOESM12_ESM.pdf]

nature portfolio

Corresponding author(s): Renier van der Hoorn

Last updated by author(s): 10.9.2023

Reporting Summary

Nature Portfolio wishes to improve the reproducibility of the work that we publish. This form provides structure for consistency and transparency in reporting. For further information on Nature Portfolio policies, see our Editorial Policies and the Editorial Policy Checklist.

Statistics

For all statistical analyses, confirm that the following items are present in the figure legend, table legend, main text, or Methods section.

n/a	Confirmed	
☐	☒	The exact sample size (*n*) for each experimental group/condition, given as a discrete number and unit of measurement
☐	☒	A statement on whether measurements were taken from distinct samples or whether the same sample was measured repeatedly
☐	☒	The statistical test(s) used AND whether they are one- or two-sided<br>*Only common tests should be described solely by name; describe more complex techniques in the Methods section.*
☐	☒	A description of all covariates tested
☐	☒	A description of any assumptions or corrections, such as tests of normality and adjustment for multiple comparisons
☐	☒	A full description of the statistical parameters including central tendency (e.g. means) or other basic estimates (e.g. regression coefficient) AND variation (e.g. standard deviation) or associated estimates of uncertainty (e.g. confidence intervals)
☐	☒	For null hypothesis testing, the test statistic (e.g. *F*, *t*, *r*) with confidence intervals, effect sizes, degrees of freedom and *P* value noted<br>*Give P values as exact values whenever suitable.*
☒	☐	For Bayesian analysis, information on the choice of priors and Markov chain Monte Carlo settings
☒	☐	For hierarchical and complex designs, identification of the appropriate level for tests and full reporting of outcomes
☒	☐	Estimates of effect sizes (e.g. Cohen's *d*, Pearson's *r*), indicating how they were calculated

Our web collection on statistics for biologists contains articles on many of the points above.

Software and code

Policy information about availability of computer code

Data collection	- Protein complexes were predicted using AlphaFold v2.1.1 (https://github.com/deepmind/alphafold/releases/tag/v2.1.1). AlphaFold was run using a custom-built workflow which can be found here: https://zenodo.org/record/7424746.<br>- To compare predicted protein structures to experimentally-resolved protein structures, we used the DALI protein server (June-July 2023), TMalign v20190425 and USalign v20220924.<br>- Small secreted proteins were predicted using a custom-made workflow that can be found here: https://zenodo.org/record/7424834, combining SignalP5.0, TargetP2.0 and ApoplastP1.0.1.<br>- For transcription analysis, we used sra-toolkit v2.10.9, centrifuge v1.0.4 with a nt nr database last updated 03/03/2018, trimmomatic v0.39 and Kallisto v0.39.<br>- To analyse P69B orthologs, we used custom-made workflows that can be accessed here: https://zenodo.org/record/7424860, https://zenodo.org/record/7424854 and https://zenodo.org/record/7424846. These pipelines combine several published tools such as trimmomatic 0.39, BWA-MEM v0.7.17, Samtools v1.7, GATK v3.8-1-0-gf15c1cef, bcftools v1.7, whatshap v1.0 and exonerate v2.4.0.<br>- All snakemake workflows were based on snakemake v6.7.0.
Data analysis	- AlphaFold output data was processed with custom python scripts, all deposited here: https://zenodo.org/record/7424746 and in supplementary File S1. We used Pymol v2.5.1.<br>- Statistical tests were run using scipy v1.11.1 and statsmodels v0.13.5.

For manuscripts utilizing custom algorithms or software that are central to the research but not yet described in published literature, software must be made available to editors and reviewers. We strongly encourage code deposition in a community repository (e.g. GitHub). See the Nature Portfolio guidelines for submitting code & software for further information.

Data

Policy information about availability of data

All manuscripts must include a data availability statement. This statement should provide the following information, where applicable:

- Accession codes, unique identifiers, or web links for publicly available datasets
- A description of any restrictions on data availability
- For clinical datasets or third party data, please ensure that the statement adheres to our policy

Source data are provided with this paper.

Research involving human participants, their data, or biological material

Policy information about studies with human participants or human data. See also policy information about sex, gender (identity/presentation), and sexual orientation and race, ethnicity and racism.

Reporting on sex and gender	N/A
Reporting on race, ethnicity, or other socially relevant groupings	N/A
Population characteristics	N/A
Recruitment	N/A
Ethics oversight	N/A

Note that full information on the approval of the study protocol must also be provided in the manuscript.

Field-specific reporting

Please select the one below that is the best fit for your research. If you are not sure, read the appropriate sections before making your selection.

☒ Life sciences ☐ Behavioural & social sciences ☐ Ecological, evolutionary & environmental sciences

For a reference copy of the document with all sections, see nature.com/documents/nr-reporting-summary-flat.pdf

Life sciences study design

All studies must disclose on these points even when the disclosure is negative.

Sample size	The competitive ABPP data of Fig. 4C was generated using a single purified P69B sample that was incubated with purified inhibitors in 3 technical replicates. The same purified SSPs were also used at 2-fold higher concentrations in 3 technical replicates, providing similar results.
Data exclusions	None
Replication	A replication experiment with independently purified proteins included 4 assays, each with n=3 technical replicates.
Randomization	Competivite ABPP assays were not randomized.
Blinding	Investigators were not blinded.

Reporting for specific materials, systems and methods

We require information from authors about some types of materials, experimental systems and methods used in many studies. Here, indicate whether each material, system or method listed is relevant to your study. If you are not sure if a list item applies to your research, read the appropriate section before selecting a response.

Materials & experimental systems

n/a	Involved in the study
x	Antibodies
x	Eukaryotic cell lines
x	Palaeontology and archaeology
x	Animals and other organisms
x	Clinical data
x	Dual use research of concern
	x Plants

Methods

n/a	Involved in the study
x	ChIP-seq
x	Flow cytometry
x	MRI-based neuroimaging
