# Supplementary material for: AlphaFold-Multimer predicts cross-kingdom interactions at the plant-pathogen interface
Source: Nat Commun. 2023 Sep 27;14:6040. doi: 10.1038/s41467-023-41721-9 (PMC10533508; doi:10.1038/s41467-023-41721-9)
Supplement: Supplementary file 13 — Source Data [file 41467_2023_41721_MOESM13_ESM.pptx]

## Slide 1
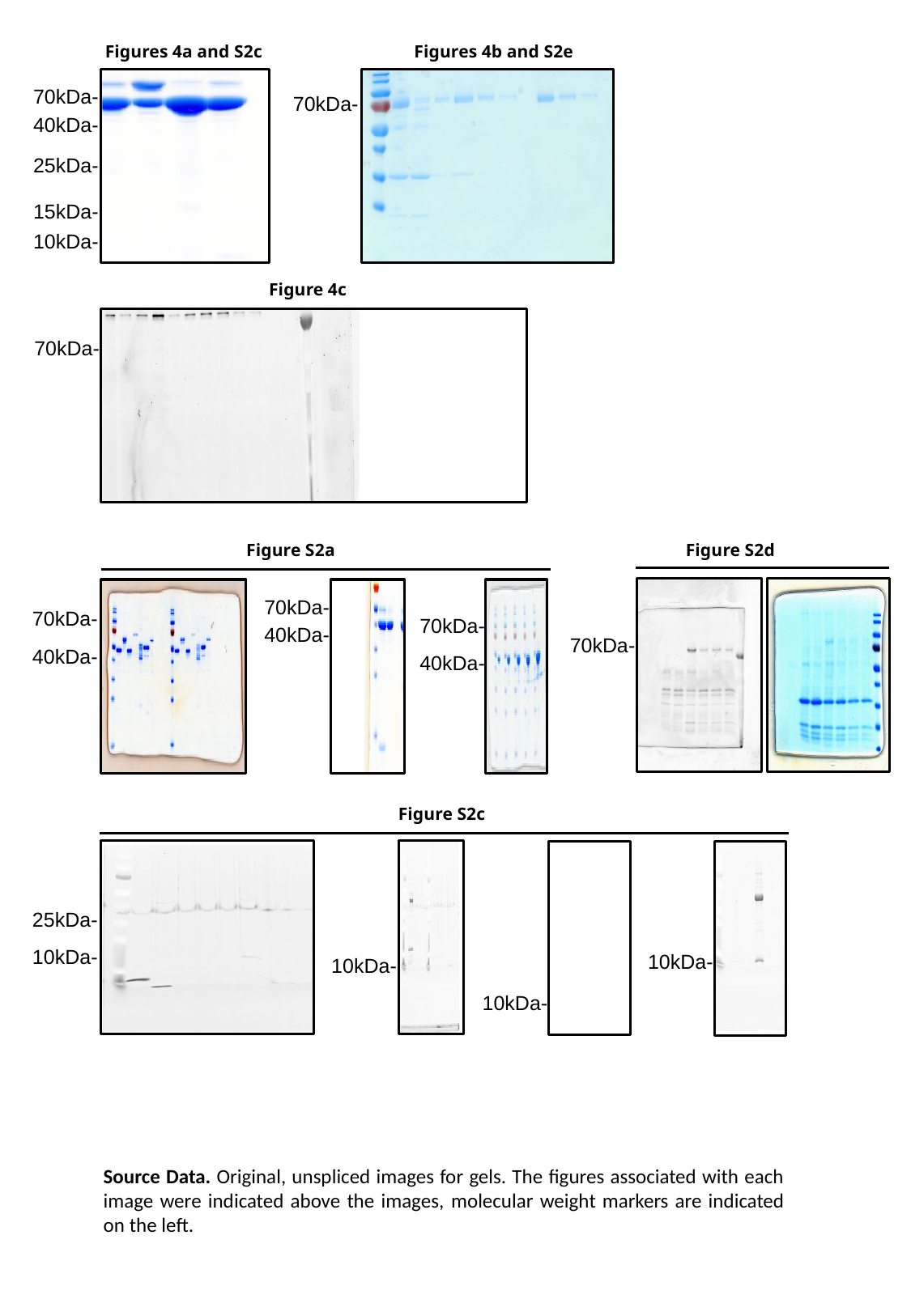

Figures 4a and S2c
70kDa-
40kDa-
25kDa-
15kDa-
10kDa-
Figures 4b and S2e
70kDa-
Figure 4c
70kDa-
Figure S2d
Figure S2a
70kDa-
40kDa-
70kDa-
40kDa-
70kDa-
40kDa-
70kDa-
Figure S2c
25kDa-
10kDa-
10kDa-
10kDa-
10kDa-
Source Data. Original, unspliced images for gels. The figures associated with each image were indicated above the images, molecular weight markers are indicated on the left.
